# Hypertension and endothelial dysfunction in the pristane model of systemic lupus erythematosus

**DOI:** 10.14814/phy2.14734

**Published:** 2021-02-01

**Authors:** Daniel M. McClung, William J. Kalusche, Katie E. Jones, Michael J. Ryan, Erin B. Taylor

**Affiliations:** ^1^ Department of Physiology and Biophysics University of Mississippi Medical Center Jackson MS USA; ^2^ G.V. (Sonny) Montgomery Veterans Affairs Medical Center Jackson MS USA

**Keywords:** autoantibodies, autoimmunity, endothelial dysfunction, hypertension, pristane, systemic lupus erythematosus

## Abstract

Autoimmune diseases such as psoriasis, rheumatoid arthritis, and systemic lupus erythematosus (SLE) have high rates of hypertension and cardiovascular disease. Systemic lupus erythematosus is a prototypic autoimmune disorder that primarily affects women of childbearing age and is associated with a loss of self‐tolerance, autoreactive B and T lymphocytes, and the production of autoantibodies, especially to nuclear components. In this study, we hypothesized that the pristane‐inducible model of SLE would develop hypertension and vascular dysfunction as the disease progressed. To test this hypothesis, female C57BL/6 mice were administered PBS or pristane. Seven months after pristane administration, mice developed various autoantibodies, including anti‐dsDNA IgG, anti‐ssDNA IgG, and anti‐nRNP IgG, as well as hypergammaglobulinemia. Several other immunological changes, including increased circulating neutrophils and increased CD4^−^CD8^−^ (double negative) thymocytes were also detected. Mean arterial pressure (MAP) was elevated in pristane‐treated mice when compared to PBS‐treated mice. In addition, second‐order mesenteric arteries from pristine‐treated mice had impaired relaxation to the endothelium‐dependent vasodilator acetylcholine compared to PBS‐treated mice. These data suggest that the immune system dysfunction present in the pristane model of lupus contributes to the development of hypertension and vascular dysfunction.

## Graphical abstract

Autoimmune diseases such as systemic lupus erythematosus have high rates of hypertension and cardiovascular disease. In this study, we induced lupus in female C57BL/6 mice with the hydrocarbon oil pristane. Seven months after pristane administration, mice developed various autoantibodies and other immunological changes indicative of lupus as well as elevated blood pressure and endothelial dysfunction in second‐order mesenteric arteries.

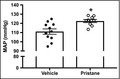

## 1 INTRODUCTION

Both clinical and experimental evidence over the past several decades implicates inflammation and immune system activation in the development and pathogenesis of hypertension (Drummond et al., 2019; Rodriguez‐Iturbe et al., 2017). For example, studies in the 1970s and 1980s identified elevated circulating IgG levels in hypertensive patients (Ebringer & Doyle, 1970; Hilme et al., 1989), and autoantibodies to the angiotensin type I receptor (Wallukat et al., 1999), heat shock proteins (Pockley et al., 2002), and the α1‐adrenergic receptor (Wenzel et al., 2008) were subsequently identified. These studies demonstrating a functional role for autoantibodies in human hypertension suggest that a loss of immunological tolerance to self‐antigens (autoimmunity), may be an important factor contributing to the development of hypertension.

In further support of the link between autoimmunity and hypertension, patients with autoimmune diseases such as psoriasis, rheumatoid arthritis (RA), and systemic lupus erythematosus (SLE) have immune system dysfunction and high rates of hypertension and cardiovascular disease (Bartoloni et al., 2018; Taylor et al., 2019). Psoriasis affects 120–180 million people worldwide, and severe psoriasis is associated with hypertension as well as an increased risk of adverse cardiovascular events (Griffiths & Barker, 2007; Mehta et al., 2011; Qureshi et al., 2009). A study by Symmons et al. found that over 50% of premature deaths in those with RA are due to CVD, and CVD is a leading cause of death in SLE, second only to mortality resulting from an active SLE flare (Trager & Ward, 2001). Because of the prevalence of hypertension and CVD in patients with autoimmune disorders, it is imperative to identify animal models that mimic these characteristics.

Several animal models of SLE are reported to develop hypertension and vascular dysfunction. Systemic lupus erythematosus is systemic autoimmune disorder that primarily affects women of childbearing age that is characterized by prominent immune system dysfunction and the development of autoantibodies to various nuclear components. The female NZBWF1 mouse model closely mimics the human disease including the genetics, environmental factors, and sex hormones (Perry et al., 2011). This model is generated by crossing a New Zealand Black mouse with a New Zealand White mouse (Andrews et al., 1978). As the mice age, they develop a lupus like syndrome. As the disease progresses, these mice develop endothelial dysfunction and increased blood pressure (Ryan & McLemore, 2007). Later studies identified roles for oxidative stress (Mathis et al., 2012), inflammatory cytokines (Venegas‐Pont et al., 2010), sex hormones (Gilbert et al., 2014), autoantibodies (Taylor et al., 2018), and B and T lymphocytes (Mathis et al., 2014, 2017; Taylor & Ryan, 2017; Taylor et al., 2019) in the development of hypertension in this model. More recently, an SLE mouse model induced by epicutaneous application of the TLR7 agonist imiquimod to BALB/c mice, was reported to develop hypertension and aortic endothelial dysfunction. The study also highlighted the importance of IL‐17 in the development of hypertension and vascular changes in this model (Robles‐Vera et al., 2020). In addition, Yan et al. demonstrated elevated blood pressure, as measured by tail cuff, in BALB/c mice administered the hydrocarbon oil pristane. The study also showed impaired endothelium‐dependent relaxation in thoracic aorta (Yan et al., 2020). While all of these models have the cardiovascular disease characteristics indicative of SLE, they differ in their various disease manifestations.

The rodent pristane‐induced model of SLE is generated by administering the hydrocarbon oil pristane into the peritoneal cavity of mice, and has been utilized to study autoimmunity since the mid‐1990s (Satoh & Reeves, 1994; Satoh et al., 1995). This model gradually leads to local chronic inflammation in the abdominal cavity; arthritis; and many features of SLE, including anti‐nuclear autoantibody production, and a type I interferon signature over a period of 5–7 months (Freitas et al., 2017). Importantly, the majority of patients with SLE have dysregulated type I interferons (Baechler et al., 2003), and other SLE models do not have this type I interferon signature (Zhuang et al., 2015). These mice also meet many of the clinical diagnostic criteria of lupus in patients, including lupus nephritis (LN), arthritis, diffuse alveolar hemorrhage, anemia, and serositis (Zhuang et al., 2015). Because of the prevalent hypertension in patients with SLE, we hypothesized that female C57BL/6 mice administered pristane would develop hypertension and vascular dysfunction in conjunction with chronic inflammation.

## 2 MATERIALS AND METHODS

### 2.1 Animals

Female C57BL/6 J mice (Jackson Laboratories, Bar Harbor, ME) were used in this study. Female mice were utilized because SLE is primarily a disease of females that affects women:men at a 9:1 ratio (Petri, 2002). In addition, the development of SLE disease has been reported to have female sex bias in the pristane inducible model (Smith et al., 2007). Mice were maintained on a 12 h light/dark cycle in temperature‐controlled rooms (73–76 degrees Fahrenheit) with access to chow and water ad libitum. All studies were performed with the approval of the University of Mississippi Medical Center Institutional Animal Care and Use Committee and in accordance with National Institutes of Health Guide for the Care and Use of Laboratory Animals.

### 2.2 Experimental protocol

At 8 weeks of age, mice were administered 0.5 ml of either pristane (*N* = 14) (2,6,10,14 Tetramethylpentadecane, Acros Organics) or PBS (*N* = 14) via intraperitoneal injection. Body weight was measured weekly to monitor animal health status. After 28 weeks, mice received carotid catheter surgery and were allowed to recover overnight. After two consecutive days of blood pressure measurement, mice were euthanized using cervical dislocation, tissues were harvested and second‐order mesenteric arteries were isolated for wire myography in *N* = 8 animals per group. A small subset of animals (*N* = 4/group) were implanted with carotid artery catheters 14 weeks after pristane administration and MAP was measured, mesenteric arteries were isolated, and vascular function measurements were performed.

### 2.3 Autoantibodies and total IgG and IgM

Anti‐dsDNA IgG, anti‐ssDNA IgG, anti‐nRNP IgG, total IgG, and total IgM were detected in plasma at 40 weeks of age (28 weeks after pristane administration) using commercially available ELISA kits (Alpha Diagnostic International, San Antonio, TX) according to the manufacturer's instructions.

### 2.4 Immunofluorescence

Autoantibodies were assessed using Hep‐2 slides (BioRad). Briefly, slides were incubated with plasma of PBS or pristane‐treated mice diluted 1:50 in PBS in a humidified chamber at room temperature for 30 min. The slides were quickly rinsed in PBS followed by two five minute washes in PBS. Goat anti‐mouse IgG FITC (Southern Biotech) at a 1:100 dilution in PBS was then added to the slides. The slides were incubated in a humidified chamber at room temperature for 30 min, protected from light. After washing two times with PBS, several drops of DAPI Fluoromount G (Southern Biotech) were added to the slides, followed by a coverslip. The slides were visualized using a Lionheart Pfx microscope (BioTek).

### 2.5 Preparation of cells for flow cytometry

Blood was collected from the retroorbital plexus at the conclusion of the study. The blood was centrifuged at 350 × g for 5 min to isolate plasma. Erythrocytes were lysed by adding 10X volume of 1X PharmLyse (BD Biosciences, San Jose, CA). After incubation for 5 min at room temperature, the blood was centrifuged at 200 × g for 5 min. The pelleted peripheral blood leukocytes (PBL) were washed with 1 X PBS, 2% FCS and centrifuged at 350 × g for 5 min. Cells were immediately used for flow cytometry. Spleens were homogenized using the Spleen Dissociation Kit (Miltenyi Biotec, Bergisch Gladbach, Germany) and the GentleMACS Octo Dissociator (Miltenyi Biotec) according to the manufacturer's instructions. For isolation of renal immune cells, one kidney was homogenized in 5 ml RPMI media containing 200 U/ml DNase and 10 mg/ml collagenase IV using the GentleMACS and a user‐defined protocol for mouse kidney. The resulting homogenate was filtered through a 70 μM cell strainer and washed with 1X PBS containing 2% FCS and 2 mM EDTA. The single‐cell suspension was centrifuged at 300 × g for 10 min. The thymus was homogenized by pressing through a 70 μM cell strainer and washing with 1X PBS, 2% FCS. The resulting single‐cell suspension was centrifuged at 350 × g for 5 min and washed one time with 10 ml of 1X PBS, 2% FCS. For spleen, kidney, and thymus, erythrocytes were lysed in 3 ml of 1X Pharm Lyse for 5 min at room temperature and washed with 27 ml of 1X PBS containing 2% FCS and 2 mM EDTA. After centrifugation at 350 × g for 5 min, the cells were used for flow cytometric analyses.

### 2.6 Flow cytometry

Flow cytometric analyses were performed as previously described in our laboratory (Taylor & Ryan, 2017). Briefly, cells were first washed and resuspended in 1X PBS, 2% FCS, and 0.9% sodium azide at a concentration of 2 × 10^7^ cells/ml. 1 × 10^6^ cells (50 μL) were aliquoted into a flow cytometry tube and incubated with 0.25 μg of anti‐mouse CD32/CD16 (FcR block, BD Biosciences) for 5 min. on ice. Cells were stained with either isotype control antibodies or antibodies shown in Table 1 for 30 min on ice protected from light. All antibodies were diluted 1:200 in 1X PBS, 2% FCS, and 0.09% sodium azide. All samples were analyzed on an LSR II flow cytometer (BD Biosciences). A total of 100,000 gated events were acquired for each sample, and data were analyzed using FCS Express 7 Software (De Novo Software).

**TABLE 1:** Antibodies used for flow cytometric analyses

Antibody	Clone	Vendor
CD45	30‐F11	BD biosciences
CD45R	RA3‐6B2	BD biosciences
CD19	1D3	BD biosciences
CD3e	17A2	BD biosciences
CD4	GK1.5	BD biosciences
CD8⍺	53–6.7	BD biosciences
TCRβ	H57‐597	BD biosciences
ɣδ TCR	GL3	BD biosciences
Ly6G	1A8	BD biosciences
Ly6C	AL−21	BD biosciences
CD11b	M1/70	BD biosciences
CD11c	HL3	BD biosciences
F4/80	BM8	Biolegend
NK1.1	PK136	BD biosciences

### 2.7 Blood pressure

Mean arterial pressure (MAP, mmHg) was recorded via indwelling carotid artery catheters in freely moving conscious mice as previously described in our laboratory (Mathis et al., 2011, 2012, 2013, 2014; Taylor & Ryan, 2017; Taylor et al., 2018; Venegas‐Pont et al., 2011). Blood pressure was measured in the morning on two consecutive days using an 8 channel PowerLab/16SP (ADInstruments) blood pressure transducer, and recorded using Chart5 for Windows (ADInstruments). A total of 90 minutes of data were recorded, with the final 60 minutes being used to determine MAP. The data for the 2 days were averaged for each mouse to determine its MAP.

### 2.8 Vascular reactivity studies

Endothelial function was assessed using wire myography. Second‐order mesenteric arteries in *N* = 8 mice were isolated at the conclusion of the study and were placed in wire myograph chambers (DMT) containing Kreb's solution (in mmol/L, pH 7.4: 118.3 NaCl, 4.7 KCl, 2.5 CaCl_2_, 1.2 MgSO_4_, 1.2 KH_2_PO_4_, 11 NaHCO_3_, 11 glucose) at 37 degrees Celsius and aerated with 95% CO_2_, 5% O_2_. The vessels were normalized by stepwise equilibration to a tension of 2.25 mN and then allowed to equilibrate for 30 min. After equilibration, the vessels were subjected to a wakeup curve consisting of 1 × 10^−5^ phenylephrine (PE) followed by 1.5 × 10^−4^ acetylcholine (Ach). Each chamber was washed four times with Kreb's solution and incubated for 15 min. After the vessels returned to baseline tension, they were subjected to a PE dose–response curve (2.5 × 10^−6^–7.5 × 10^−5 ^M). The wash and incubation steps were repeated as described earlier, and then concentration‐dependent relaxation (10^−8^–10^−4 ^M) to Ach and sodium nitroprusside (SNP) was assessed in vessels precontracted with PE.

### 2.9 Renal injury

Glomerulosclerosis was assessed in *N* = 8 mice from each group using hematoxylin and eosin staining as previously described by our laboratory (Mathis et al., 2014). To determine the glomerulosclerosis index, a total of 50 glomeruli were scored blinded using a scale of 0–4: 0: no sclerosis, 1: up to 25% of the glomerulus is sclerosed, 2: 26–50% of the glomerulus is sclerosed, 3: 51–75% of the glomerulus is sclerosed, 4: 76–50% of the glomerulus is sclerosed. The score was tabulated by multiplying each score by the number of glomeruli with that score divided by the total number scored. Plasma blood urea nitrogen was assessed in 6 μl of plasma using a Vet Axcel Chemistry Analyzer (Alfa Wasserman, Diagnostic Technologies, LLC). In a subset of animals (*N* = 8 from each group), urinary albumin was assessed using an Albumin ELISA (Alpha Diagnostic) according to the manufacturer's instructions.

### 2.10 Statistical analysis

Data are presented as mean ± SEM. Statistical analyses were performed using GraphPad Prism 7. An unpaired *t*‐test was used to analyze differences between mice administered PBS or pristane. A two‐way ANOVA with repeated measures was used to test between significant differences in dose–response curves in the vascular studies. A *p* < 0.05 was considered statistically significant.

## 3 RESULTS

**FIGURE 1:**
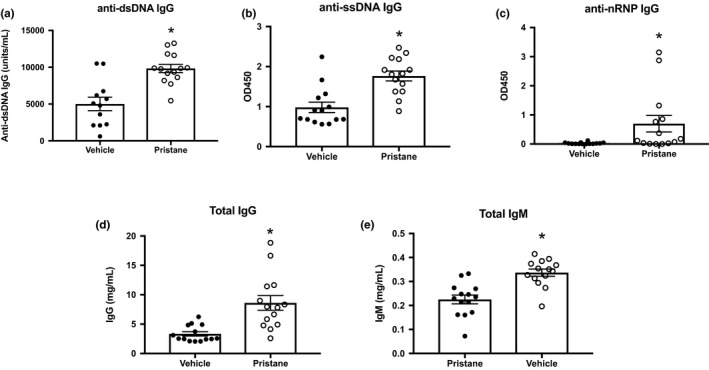
C57BL/6 mice administered pristane develop autoantibodies and elevated IgG and IgM levels. Autoantibody levels and total IgG and IgM concentrations were assessed using ELISA in plasma of PBS (*N* = 12) and pristane‐treated (*N* = 14) mice 28 weeks after treatment. (a) anti‐dsDNA IgG, (b) anti‐ssDNA IgG, (c) anti‐nRNP IgG, (d) total IgG, (e) total IgM. *, *p* < 0.05 versus PBS‐treated mice by *t*‐test

Because pristane has been shown to induce autoantibodies to nucleic acids and nucleic acid‐associated proteins, we analyzed autoantibody production in PBS‐ and pristine‐treated mice. Mice administered pristane have increased levels of anti‐ssDNA, anti‐dsDNA, and anti‐rRNP autoantibodies as compared to PBS‐treated mice (Figure 1(a)–(c), *p* < 0.05 vs. PBS). In addition, pristane‐treated mice have increased circulating IgG and IgM (Figure 1(d)–(e), *p* < 0.05 vs. PBS). Autoantibody production was also assessed using Hep‐2 slides. The PBS‐treated mice exhibited little to no autoantibody staining on the Hep‐2 slides (Figure S1(a)), whereas mice treated with pristane developed distinct staining profiles, including homogenous nuclear staining, cytoplasmic staining, and speckled staining (Figure S1(b)–(d)).

**FIGURE 2:**
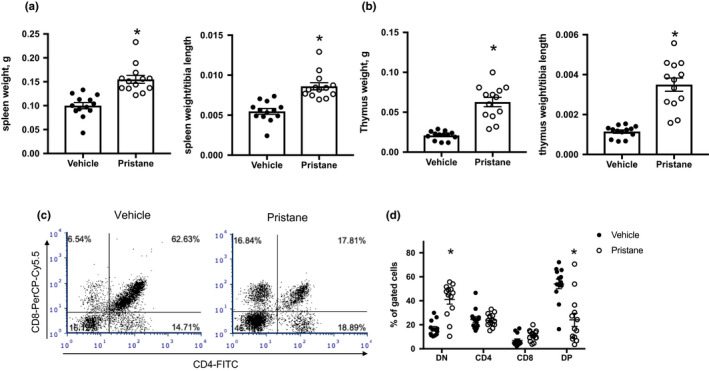
Autoimmunity results in splenomegaly and altered thymic cell populations. (a) Spleen weight and spleen weight/tibia length. (b) Thymic weight and thymic weight/tibia length. (c) Representative dot plot of thymocytes stained with anti‐CD4‐FITC and anti‐CD8‐PerCP‐Cy5.5. (d) Percentages of double‐negative (CD4^−^CD8^−^), single‐positive (CD4^+^ or CD8^+^), and double‐positive (CD4^+^CD8^+^) thymocytes in PBS and pristane mice. PBS‐treated animals, *N* = 14; Pristane‐treated animals, *N* = 14. *, *p* < 0.05 versus PBS for each cell population by *t*‐test

**FIGURE 3:**
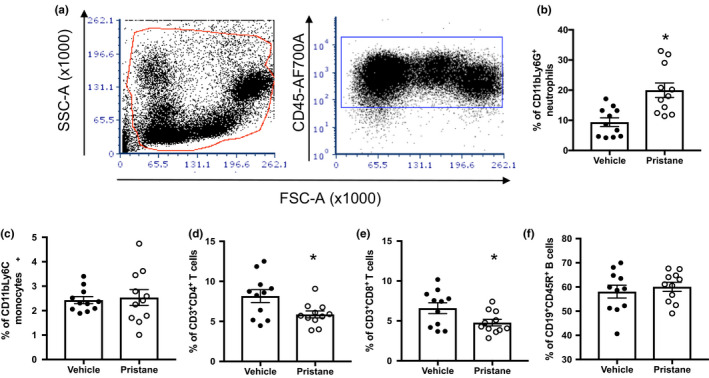
Circulating leukocyte populations are altered in C57BL/6 mice treated with pristane. (a) Representative gating strategy to identify PBL, (b) Percentage of CD11b^+^Ly6G^+^ neutrophils, (c) Percentage of CD11b^+^Ly6C^+^ monocytes, (d) Percentage of CD3^+^CD4^+^ T cells, (e) Percentage of CD3^+^CD8^+^ T cells, (f) Percentage of CD19^+^CD45R^+^ B cells. PBS‐treated animals, *N* = 14; Pristane‐treated animals, *N* = 14. *, *p* < 0.05 versus PBS‐treated mice by *t*‐test

As markers of immune activity, spleen weight and thymus weight were recorded at tissue harvest. Mice administered pristane displayed splenomegaly and increased thymus weight when compared with PBS‐treated animals (*p* < 0.05, Figure 2(a)–(b)). Flow cytometric analyses of thymocytes revealed the increased levels of CD4^−^CD8^−^ double‐negative thymocytes and decreased levels of CD4^+^CD8^+^ double‐positive thymocytes in mice administered pristane (Figure 2(c)–(d)), indicating an impairment in the transition from double‐negative to double‐positive thymocytes. Splenocytes were also analyzed for changes in immune cell populations; however, no significant differences in the relative percentages of macrophages, monocytes, neutrophils, T cells, B cells, or NK cells were found (data not shown). Finally, PBL were analyzed at the conclusion of the study (Figure 3), and pristane mice had increased percentages of CD11b^+^Ly6G^+^ neutrophils and decreased percentages of both CD4^+^ and CD8^+^ T cells. There were no significant differences in monocytes or B cells.

**FIGURE 4:**
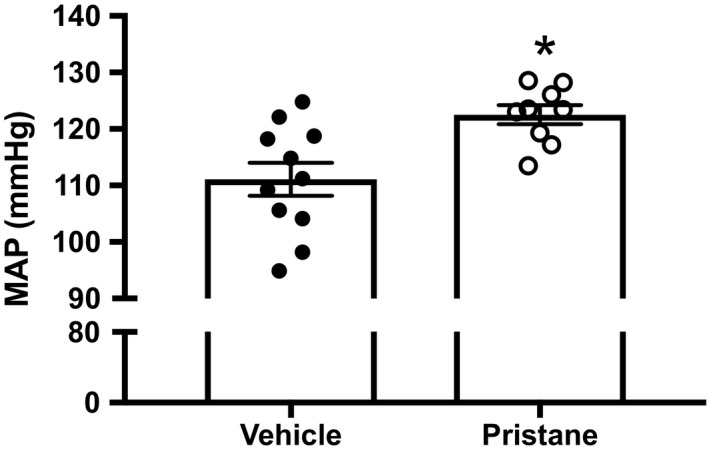
Mean arterial pressure is increased in mice administered pristane after 28 weeks. Mean arterial pressure in mice 28 weeks after pristane administration. PBS‐treated animals, *N* = 11; Pristane‐treated animals, *N* = 9 for 28 weeks. PBS*, *p* < 0.05 versus PBS‐treated mice by *t*‐test

To analyze the development of hypertension in an inducible model of SLE, we measured MAP in conscious freely moving mice 28 weeks after pristane treatment. Mice administered pristane have elevated blood pressure compared to PBS‐treated mice (*p* < 0.05, Figure 4(a)). We also recorded MAP in a separate set of PBS‐ and pristine‐treated mice 14 weeks after treatment and found no difference in MAP (Figure S2), suggesting that blood pressure increases as SLE disease develops.

**FIGURE 5:**
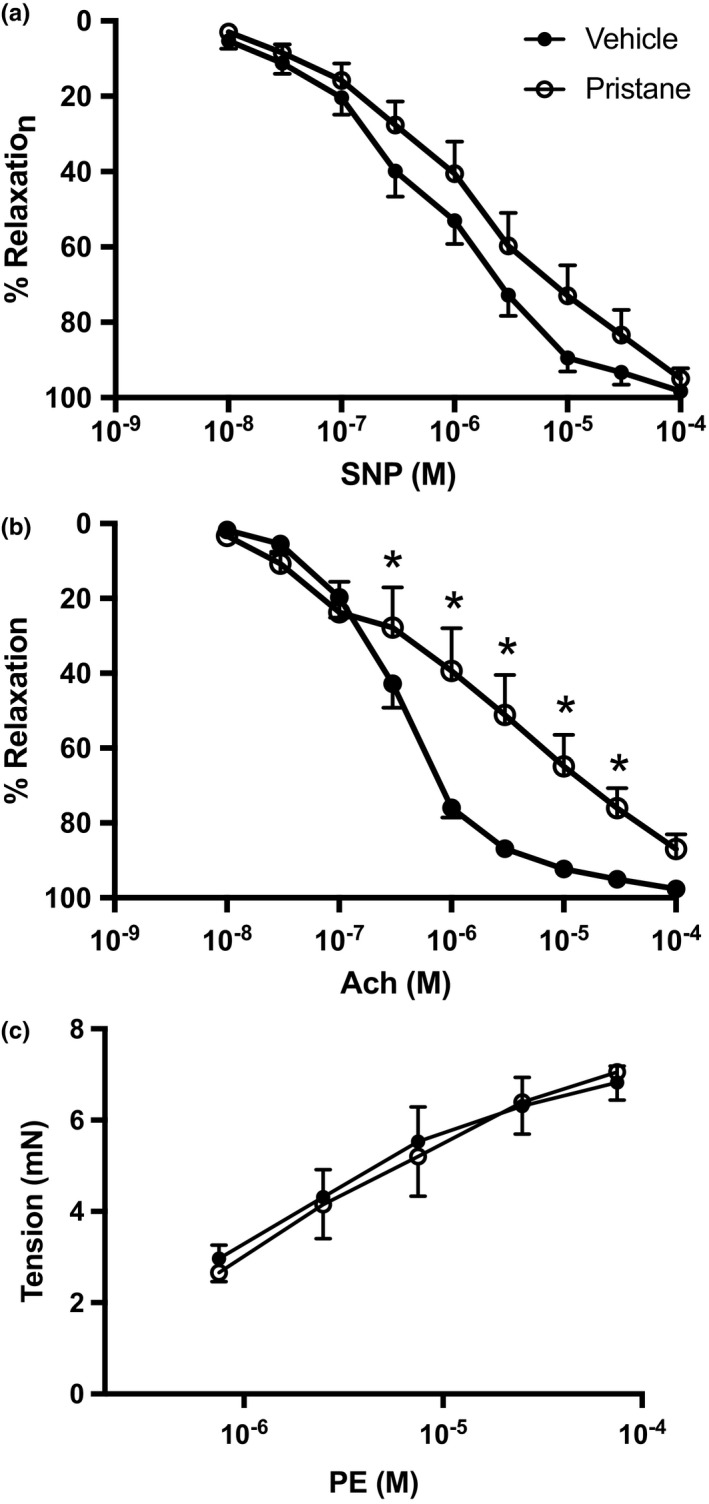
Pristane mice have endothelial dysfunction 28 weeks after pristane administration. Second‐order mesenteric arteries were isolated and vascular function was assessed. (a) Sodium nitroprusside dose–response curve, (b) Ach dose–response curve, (c) PE dose–response curve at 28 weeks. PBS‐treated animals, *N* = 14; Pristane‐treated animals, *N* = 14. *, *p* < 0.05 versus. PBS‐treated mice by two‐way repeated measures ANOVA

To identify whether vascular changes were also present in this model, vascular function was assessed in second order mesenteric arteries precontracted with PE. As shown in Figure 5(a), endothelium‐independent relaxation, as assessed with SNP, is not different between PBS and pristane‐treated mice. However, pristane‐treated mice developed endothelial dysfunction, as shown by impaired relaxation to increasing concentrations of the endothelium‐dependent vasodilator Ach (*p* < 0.05, Figure 5(b)). The logEC50 values of Ach are also significantly different between PBS and pristane mice (Table 2). We also examined dose responses to increasing concentrations of PE and found no differences in the contractility curves in PBS and pristane mice (Figure 5(c)). At 14 weeks after pristane treatment, mice display no evidence of endothelial dysfunction (Figure S3), suggesting that similar to the development of hypertension, endothelial dysfunction develops as the disease progresses.

**TABLE 2:** logEC50 values for concentration–response curves

	Control	Pristane
Ach	6.45 ± 0.05	4.71 ± 0.57*
SNP	6.15 ± 0.16	5.68 ± 0.29
PE	5.81 ± 0.82	5.28 ± 0.60

* *p* < 0.05 versus control.

**FIGURE 6:**
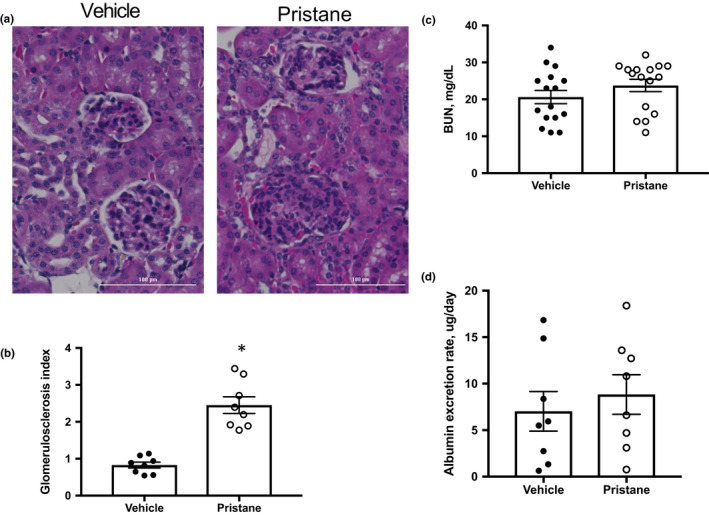
Pristane‐treated mice develop glomerulosclerosis. (a) Representative images of glomerulosclerosis (20X) from paraffin‐embedded kidneys stained with H&E, (b) Glomerulosclerosis index, (c) Blood urea nitrogen as assessed using a Vet Axcel Chemistry Analyzer. (d) Urine albumin excretion, as measured by an albumin ELISA at the conclusion of the study. PBS‐treated animals, *N* = 8; Pristane‐treated animals, *N* = 8. *, *p* < 0.05 versus PBS‐treated mice by *t*‐test

**FIGURE 7:**
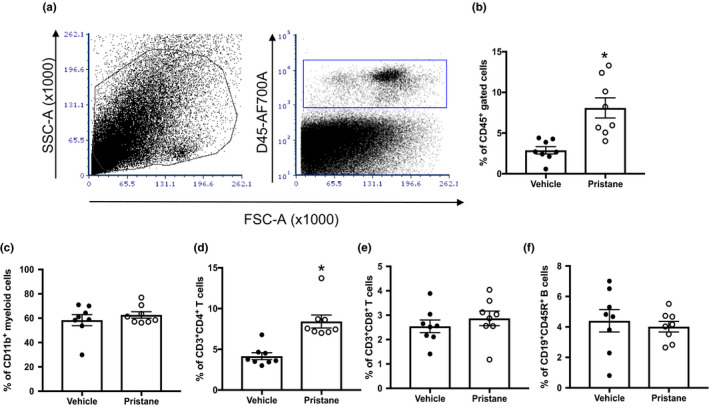
Mice administered pristane have increased renal immune cell infiltration. (a) Representative gating strategy to identify renal immune cells, (b) Percentage of CD45^+^ cells among gated renal cells, (c) Percentage of CD11b^+^ myeloid cells (d) Percentage of CD3^+^CD4^+^ T cells, (e) Percentage of CD3^+^CD8^+^ T cells, (f) Percentage of CD19^+^CD45R^+^ B cells. PBS‐treated animals, *N* = 8; Pristane‐treated animals, *N* = 8. *, *p* < 0.05 versus PBS‐treated mice by *t*‐test

Finally, we tested the effect of pristane on renal injury. Glomerular injury was assessed in hematoxylin‐ and eosin‐stained kidney sections. Mice treated with pristane exhibited mesangial hypercellularity (Figure 6(a)), which was previously reported by others utilizing the C57BL/6 mouse in pristane‐induced models of SLE (Reeves et al., 2009), in addition to a significantly increased glomerulosclerosis index (Figure 6(b)). We also measured blood urea nitrogen (BUN) and urinary albumin excretion for additional measures of renal injury. There were no significant differences in either BUN or albuminuria between PBS and pristane mice (Figure 6(c)–(d)). Renal immune cell infiltration was assessed using flow cytometry (Figure 7). Pristane mice had increased percentages of CD45^+^ immune cells in their kidneys (Figure 7(b)) and increased renal CD4^+^ T cells (Figure 7(d)).

## 4 DISCUSSION

Patients with autoimmune diseases such as SLE have an increased prevalence of hypertension and cardiovascular disease, and immune system dysfunction is implicated in the development of these complications. In this study, we sought to analyze blood pressure, vascular function, and renal injury in an inducible model of SLE, the pristane model. The major findings of the current study are the following: (1) female C57BL/6 J mice administered pristane develop hypertension, (2) mice administered pristane develop endothelial dysfunction in small high resistance arteries, and (3) pristane mice have variable renal injury, highlighted by glomerulosclerosis and immune cell infiltration but no changes in urinary albumin excretion or BUN. These findings advance our understanding of the development of hypertension and vascular dysfunction in patients with autoimmune disorders.

It has been widely reported that patients with SLE have increased blood pressure, although the prevalence depends on the cohort examined (Taylor et al., 2019). The prevalent hypertension is especially remarkable in women under the age of 40; one study reported a prevalence of 40% compared to 11% in age‐matched controls (Sabio et al., 2011). Mice administered pristane had elevated blood pressure 28 weeks after pristane treatment compared to PBS‐treated mice, but showed no changes in MAP 14 weeks after pristane treatment (Figure S2), suggesting that the development of hypertension tracked with the development of the disease. We also assessed vascular function and renal injury, two important determinants of blood pressure. The presence of endothelial dysfunction in mice treated with pristane in mesenteric arteries is important, because the mesenteric bed consists of high conductance arteries that are an important contributor to total peripheral resistance (Christensen & Mulvany, 1993) and the development of hypertension (Schiffrin, 1992). To our knowledge, this is the first assessment of vascular function using mesenteric arteries in a rodent model of autoimmunity. We did not find evidence of endothelial dysfunction at 14 weeks after pristane administration (Figure S3); therefore, it is difficult to determine whether the increased pressure caused the endothelial dysfunction or was preceded by it. In comparison, the female NZBWF1 mouse model of SLE exhibits endothelial dysfunction by 20 weeks of age, but does not have elevated blood pressure compared to control NZW mice at the same time point (Ryan & McLemore, 2007).

Several markers of renal injury were assessed in the present study, including glomerulosclerosis, albuminuria, BUN, and renal immune cell infiltration. Studies using BALB/c mice injected with pristane develop immune complex mediated glomerulonephritis, a hallmark characteristic of SLE (Satoh et al., 1995). Other strains such as the SJL mice also develop severe glomerulonephritis (Satoh et al., 1996), but the C57 strains (used here) exhibit a milder nephritis characterized by increases in mesangial matrix and cellularity indicative of Class II Lupus nephritis (Chowdhary et al., 2007). Mesangial hypercellularity can be seen in the representative histology images in Figure 6. Neither BUN nor urinary albumin excretion were increased in our study (Figure 6). This is in contrast to published studies reporting increased urinary albumin and protein excretion in pristane mice, especially BALB/c (Kienhofer et al., 2017; Richards et al., 2001). Most researchers report a more modest increase in proteinuria in C57BL/6 mice (Urbonaviciute et al., 2013); however, urinary albumin is not frequently measured. One study did report albuminuria in C57BL/6 mice 40 weeks after pristane administration (Luo et al., 2016). Consistent with our studies in the NZBWF1 model, as well as previous studies in pristane‐induced lupus (Lech & Anders, 2013; Summers et al., 2014; Taylor et al., 2018; Taylor & Ryan, 2017), we detected increased CD45^+^ and CD4^+^ T cells in the kidney. Renal immune cell infiltration may mechanistically contribute to the hypertension in this model, with CD4^+^ T cells contributing to tissue inflammation in the kidneys by secreting cytokines. Both Th1 and Th17 cells are reportedly involved in the pathogenesis of renal injury in animal models of autoimmunity (Paust et al., 2009; Phoon et al., 2008). However, it is important to note that recent studies in the MRL/*lpr* model of lupus nephritis showed that infiltrating T cells have an ‘exhausted’ phenotype characterized by reduced cytokine production and proliferation (Tilstra et al., 2018). Thus, it will be important to further phenotype the T‐cell subsets within the kidneys of these mice.

We also analyzed autoantibodies and immunoglobulins as well as immune cell populations in the circulation, spleen, and thymus. We identified several immunological changes that could potentially contribute to the hypertension, vascular dysfunction, and/or renal injury. These include increased circulating autoantibodies and immunoglobulins, increased circulating neutrophils, and changes within the thymus. We analyzed various autoantibodies (Figure 1 and Figure S1), and found that pristane‐treated mice have elevated circulating anti‐dsDNA, anti‐ssDNA, and anti‐nRNP IgG. The Hep‐2 slides, which are used clinically for anti‐nuclear antibody (ANA) analyses, also revealed other autoantibodies that are likely to be variably present in pristane mice such as anti‐chromatin antibodies, anti‐histone antibodies, and antibodies to various cytoplasmic components, as demonstrated by the cytoplasmic staining in Figure S1(b). These mice also had increased circulating IgG and IgM. Some patients with essential or refractory hypertension have elevated circulating immunoglobulins, and several autoantibodies have are reported to have a role in blood pressure control, including the AT_1_R, α_1_‐adrenergic receptor, l‐type voltage‐gated calcium channels, and heat shock protein 70 (Chan et al., 2014). Some of these antibodies, including agonistic AT_1_R autoantibodies, are reportedly present in patients with SLE. This autoantibody was associated with microvascular damage in patients with lupus nephritis, but did not correlate with increased blood pressure in this cohort (Mejia‐Vilet et al., 2020). In contrast, another study reported a correlation between blood pressure and the presence of the AT_1_R autoantibody (Xiong et al., 2013). Unfortunately, little is known about the presence of these antibodies in mouse models of SLE; however, the presence of these autoantibodies, especially in hypertensive models of autoimmunity, warrants further investigation.

Other nuclear and cytoplasmic autoantibodies can potentially affect blood pressure, vascular function, and renal injury because of their pathogenic actions, including immune complex formation and deposition, direct binding to cell surface receptors and proteins, binding to apoptotic cells, and binding to cross‐reactive extracellular molecules (Rekvig et al., 2012). Immune complex formation is likely an important factor in vascular injury, because autoantibody immune complexes deposit in endothelial basement membranes, resulting in monocyte activation, increased immune cell adhesion, and a proinflammatory environment in the vasculature. Anti‐dsDNA autoantibodies are involved in the development of lupus nephritis, namely by binding to antigens on resident renal cells such as annexin II and alpha‐actinin or binding to extracellular matrix components. This can cause renal inflammation and fibrosis (Yung & Chan, 2015). While less is known about the pathogenic role of anti‐ssDNA autoantibodies, early studies showed that polyclonal anti‐ssDNA antibodies can bind to HUVEC cells and increase the expression of leukocyte adhesion molecules (Chan et al., 1995). Also, a heterogeneous population of autoantibodies, termed anti‐endothelial cell antibodies, are prevalent in SLE patients and have the potential to cause damage to the vascular endothelium. A clinical study determined that increased levels of these antibodies correlate with renal injury and vascular damage (Perry et al., 1993), and in vitro investigations demonstrated that this autoantibody population can enhance leukocyte adhesion to endothelial cells (Florey et al., 2007).

We also identified increased percentages of CD11b^+^Ly6G^+^ neutrophils in the peripheral blood (Figure 3). Neutrophils are central in SLE disease pathogenesis, both as effector cells in inflammation, and a source of autoantigens through neutrophil extracellular trap (NET)osis, or a specialized form of cell death in which decondensed chromatin is released into the extracellular space, in addition to other proteins and molecules normally sequestered within the cell. Patients with SLE have both increased NETosis and decreased NET degradation, thus leading to increased exposure to the nuclear antigens and potentiating autoantibody production (Salemme et al., 2019). While there are limited data functionally linking neutrophils to the development of hypertension, several clinical studies have shown that an increased neutrophil to lymphocyte ratio (NLR) in the peripheral blood is associated with the development of hypertension (Liu et al., 2015), and that patients with established essential hypertension have an increased NLR (Belen et al., 2015). Elegant *in vitro* studies using sera from SLE patients uncovered a mechanism whereby NET formation leads to increased extracellular matrix metalloproteinase (MMP)‐9, which subsequently activates MMP‐2 in endothelial cells leading to increased endothelial cell death and vascular dysfunction (Carmona‐Rivera et al., 2015).

For a measure of T‐cell development and selection, we analyzed the thymus in PBS‐ and pristine‐treated mice. The mice administered pristane have larger thymuses, and we also identified decreased relative percentages of double‐positive thymocytes and increased percentages of double‐negative thymocytes (Figure 2). Under normal conditions, the majority of thymocytes are double positive (Scollay et al., 1984). Similar increases in double‐negative thymocytes has been demonstrated in the MRL/*lpr* and BXSB models of SLE (Kakkanaiah et al., 1990). Subsequent studies in the MRL/*lpr* mouse led to the conclusion that the double‐negative T cells that accumulate in the periphery migrate from the thymus (Kakkanaiah et al., 1991). The significance of this finding in the pristine‐treated mice remains unclear, due to the paucity of data regarding the thymus in the pristane model of SLE. It is possible, however, that this change promotes the escape of autoreactive T cells into the periphery, contributing to autoimmune pathology and potentially the development of vascular dysfunction and hypertension.

In conclusion, hypertension and CVD are major complications in autoimmune diseases such as SLE. This study demonstrates that, similar to the NZBWF1 mouse and the imiquimod‐inducible model, C57BL/6 mice treated with pristane develop hypertension and vascular dysfunction. The pristane model recapitulates type I interferon signature, and has many immunological changes that may be involved in the pathophysiology of these disease complications. Thus, the model of pristane‐induced SLE provides an opportunity to elucidate new mechanisms that contribute to the development of vascular dysfunction and hypertension in SLE.

## CONFLICT OF INTEREST

The authors declare no conflict of interest.

## AUTHORS’ CONTRIBUTIONS

DMM and EBT conceived and designed experiments, DMM, WJK, KEJ, and EBT performed experiments, DMM, WJK, KEJ, and EBT analyzed data, MJR and EBT interpreted data, DMM and EBT prepared figures, EBT drafted manuscript, EBT and MJR edited and revised manuscript, and DMM, WJK, KEJ, MJR, and EBT approved the final version of the manuscript.

## Supporting information



Fig S1‐S3
