# Supplementary figures and images for: Hypertension and endothelial dysfunction in the pristane model of systemic lupus erythematosus
Source: Physiol Rep. 2021 Feb 1;9(3):e14734. doi: 10.14814/phy2.14734 (PMC7851437; doi:10.14814/phy2.14734)

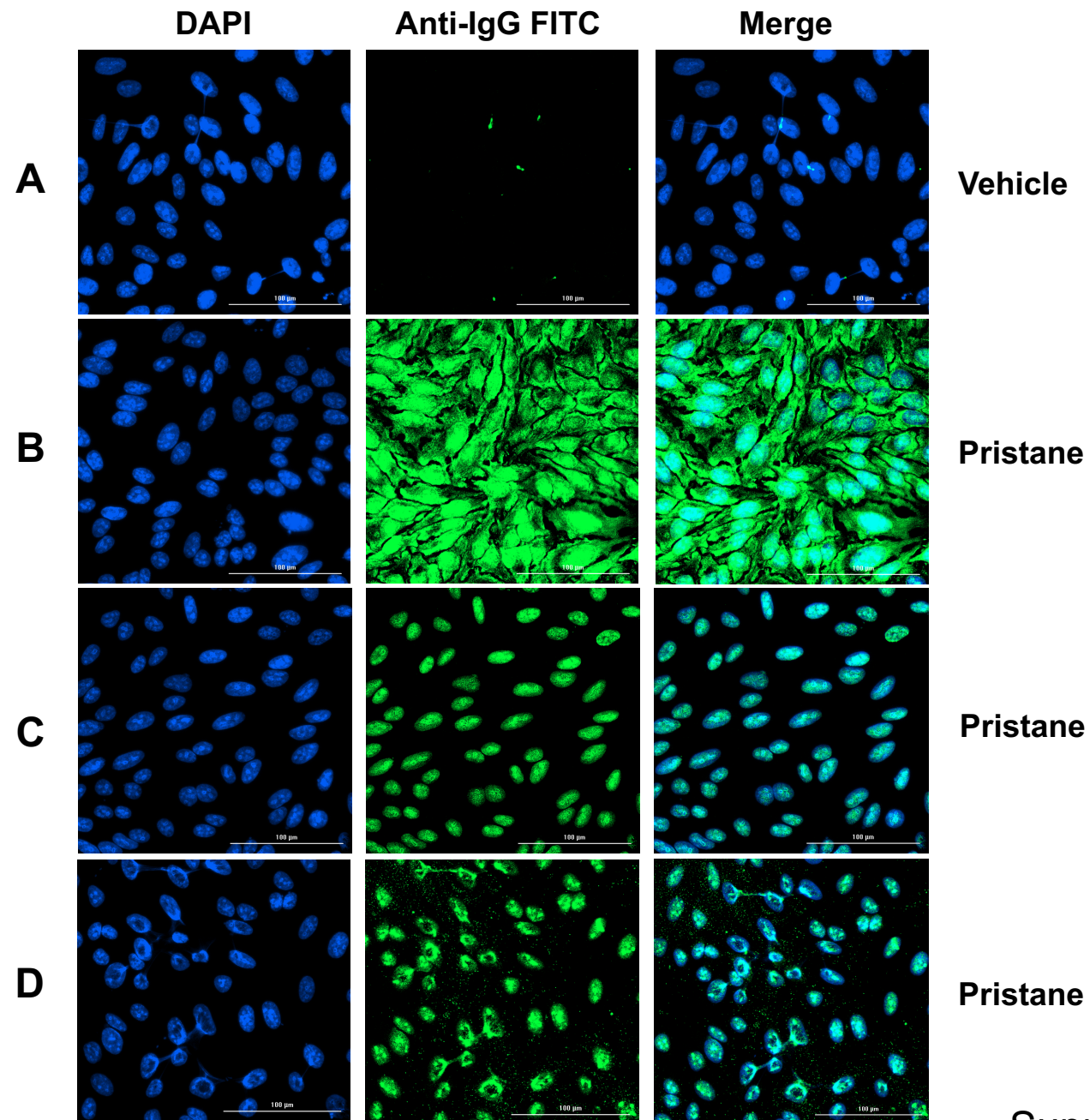

Supplemental Figure 1

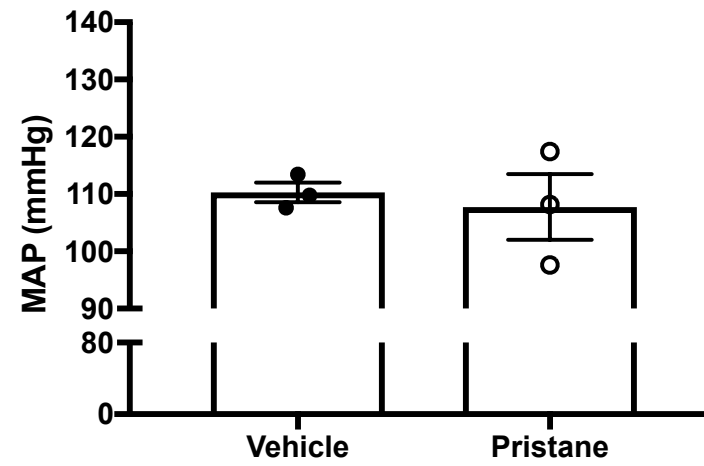

Supplemental Figure 2

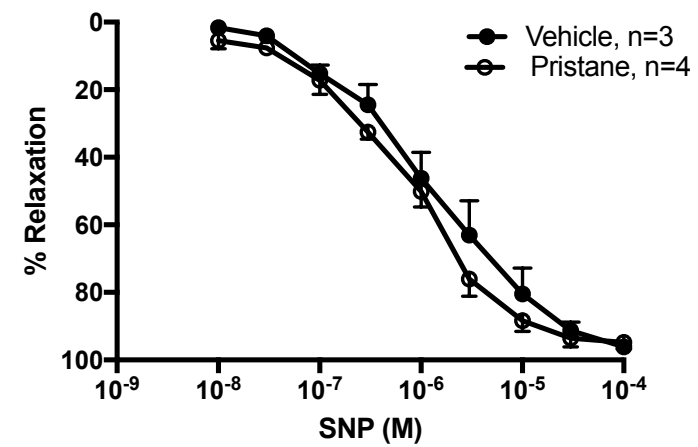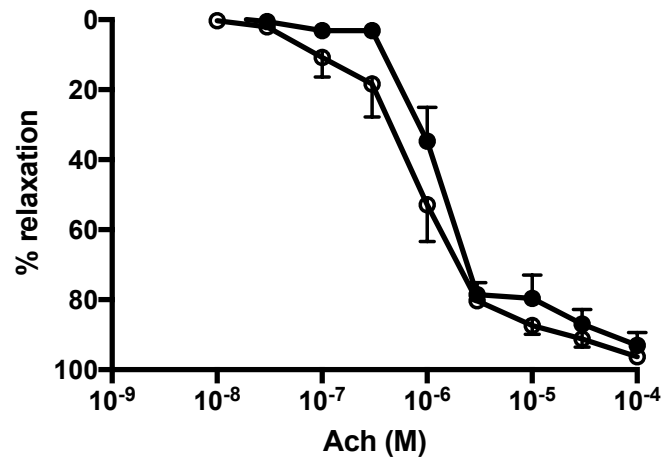

Supplemental Figure 3

Supplement: Supplementary file 1 — Fig S1‐S3 [file PHY2-9-e14734-s001.pdf]
